# Medullary carcinoma of the thyroid: an update

**DOI:** 10.1186/1756-6614-6-S2-A2

**Published:** 2013-04-05

**Authors:** Maria Alevizaki

**Affiliations:** 1 Endocrine Unit, Dept Medical Therapeutics, Athens University School of Medicine, Athens, Greece

## 

Medullary carcinoma of the thyroid (MTC) represents 5-10% of differentiated thyroid cancers. It derives from the thyroid C-cells which produce calcitonin and are of neuroendocrine origin. It is a rather rare tumour and is accompanied by considerable mortality as it has frequently already metastasised at diagnosis and is not sensitive to either radiation or chemotherapy. Recently it has been shown that MTC is not infrequent in multinodular thyroid disease and thus universal calcitonin screening has been suggested in the diagnostic workup of thyroid nodules. The prognosis of the disease has recently improved as many of the newly diagnosed cases are now small, non-invasive tumours, that probably were previously missed.

MTC is an interesting tumour as it occurs in a familial form in >25% of the cases, as part of the multiple endocrine neoplasia 2 syndromes (MEN2). When familial MTC may occur in combination with pheochromocytomas and, more rarely, with primary hyperparathyroidism. Recent advances in MTC management include preclinical diagnosis using molecular techniques in the case of familial disease and prophylactic thyroidectomy in asymptomatic gene carriers. The gene responsible for transmission of the MEN2 is the ret proto-oncogene, which is a membrane receptor tyrosine kinase; its physiological role is the transmission of neural signals during development. Most of the mutations which are found in MEN2 syndromes are clustered in the cysteine rich extracellular domain of the protein and either cause dimerisation or ligand-independent activation. Novel previously unknown ret mutations across many regions of the gene are currently being recognized. Mutation analysis should be performed in all cases of MTC because these sometimes represent unrecognized familial disease. When a mutation is identified, family members are screened and carriers are offered prophylactic thyroidectomy at a young age. The type of mutation is classified into more or less aggressive and it is important for the penetrance of the disease. The majority of experts suggest prophylactic surgery at 5-10 years, especially when the specific mutation associates with risk for earlier MTC development and more aggressive disease. Several recent publications have examined the success/cure rate of prophylactic treatment.

One important development in the field of MTC concerns the use of newer antineoplastic agents, including agents targeting the molecular pathways involved in its pathogenesis. Novel multi targeted tyrosine kinase inhibitors are being tested in advanced disease in the context of clinical trials. Recent publications indicate that a substantial proportion of patients with advanced disease will stabilize with the use of these promising agents. Patients with familial disease appear to respond better to therapy with tyrosine kinase inhibitors. Combination therapies are also being tested.

